# scAnnotate: an automated cell-type annotation tool for single-cell RNA-sequencing data

**DOI:** 10.1093/bioadv/vbad030

**Published:** 2023-03-13

**Authors:** Xiangling Ji, Danielle Tsao, Kailun Bai, Min Tsao, Li Xing, Xuekui Zhang

**Affiliations:** Department of Mathematics and Statistics, University of Victoria, Victoria V8P 5C2, Canada; Department of Mathematics and Statistics, University of Victoria, Victoria V8P 5C2, Canada; Department of Mathematics and Statistics, University of Victoria, Victoria V8P 5C2, Canada; Department of Mathematics and Statistics, University of Victoria, Victoria V8P 5C2, Canada; Department of Mathematics and Statistics, University of Saskatchewan, Saskatoon S7N 5C9, Canada; Department of Mathematics and Statistics, University of Victoria, Victoria V8P 5C2, Canada

## Abstract

**Motivation:**

Single-cell RNA-sequencing (scRNA-seq) technology enables researchers to investigate a genome at the cellular level with unprecedented resolution. An organism consists of a heterogeneous collection of cell types, each of which plays a distinct role in various biological processes. Hence, the first step of scRNA-seq data analysis is often to distinguish cell types so they can be investigated separately. Researchers have recently developed several automated cell-type annotation tools, requiring neither biological knowledge nor subjective human decisions. Dropout is a crucial characteristic of scRNA-seq data widely used in differential expression analysis. However, no current cell annotation method explicitly utilizes dropout information. Fully utilizing dropout information motivated this work.

**Results:**

We present scAnnotate, a cell annotation tool that fully utilizes dropout information. We model every gene’s marginal distribution using a mixture model, which describes both the dropout proportion and the distribution of the non-dropout expression levels. Then, using an ensemble machine learning approach, we combine the mixture models of all genes into a single model for cell-type annotation. This combining approach can avoid estimating numerous parameters in the high-dimensional joint distribution of all genes. Using 14 real scRNA-seq datasets, we demonstrate that scAnnotate is competitive against nine existing annotation methods. Furthermore, because of its distinct modelling strategy, scAnnotate’s misclassified cells differ greatly from competitor methods. This suggests using scAnnotate together with other methods could further improve annotation accuracy.

**Availability and implementation:**

We implemented scAnnotate as an R package and made it publicly available from CRAN: https://cran.r-project.org/package=scAnnotate.

**Supplementary information:**

Supplementary data are available at *Bioinformatics Advances* online.

## 1 Introduction

Many biological processes in the human body rely on the coaction of numerous cell types, each with its own designated function. Cell identification is thus crucial in studying biological phenomena and developing medical practices; pathology, for example, hinges on the accuracy of this task. Although standard immunophenotyping methods are widely practiced for cell identification, their heavy reliance on the manual selection of antibodies, markers and fluorochromes renders new and rare cell types particularly difficult to identify (Gown, 2008; Leach *et al.*, 2013). Conversely, newly developed single-cell RNA sequencing (scRNA-seq) technologies (Tang *et al.*, 2009) have heightened the detail with which we can examine cell composition by offering an unprecedented resolution of gene expression at the cellular level. The recent surge of available scRNA-seq data allows for increased accuracy in several aspects of genomic data analysis, including cell annotation (Artegiani *et al.*, 2017; Chen *et al.*, 2019; Diaz-Mejia *et al.*, 2019).

Cell-type annotation using scRNA-seq data enables researchers to distinguish various types of cells from heterozygous populations, then investigate each cell type separately and learn their interactions. Hence, cell-type annotation is often the first step of scRNA-seq data analysis, which has led to a recent surge of methods developed for this task. Pasquini *et al.* (2021) discuss 24 scRNA-seq cell-type annotation methods developed in the last 5 years. The most popular cell-type annotation approach was clustering analysis followed by manual annotation. The most important advantage of such an approach is that it does not require training a model using another ‘annotated’ scRNA-seq dataset. However, such unsupervised machine learning approaches have a critical issue; namely, they require users to manually label the cell types for each cluster of cells. The manual decisions need special biological knowledge and are subjective to researchers’ individual opinions, which can be time-consuming and inconsistent. In the last few years, a huge amount of scRNA-seq data were generated and made publicly available. These rich resources made it easier and easier to identify suitable data for training supervised machine learning models to annotate new scRNA-seq data. Recently, many supervised machine learning methods have been developed for cell-type annotation.

Currently, the discriminative classification approach dominates supervised machine learning methods for cell-type annotation. This discriminative classification approach models the distribution of cell types conditional on genomic data. For example, CaSTLe (Lieberman *et al.*, 2018) employs an XGBoost (Chen and Guestrin, 2016) classification model and SingleCellNet (Tan and Cahan, 2019) trains a Random Forest classifier on discriminating gene pairs. CHETAH (de Kanter *et al.*, 2019) and scClassify (Lin *et al.*, 2020) construct hierarchical classification trees and evaluate the correlation of query cells to reference cell types or apply an ensemble of weighted K-Nearest Neighbors Algorithm (kNN) classifiers, respectively. In SingleR (Aran *et al.*, 2019), the Spearman rank correlations of query cells to reference samples are used in an altered kNN classification algorithm. Similarly, scmap (Kiselev *et al.*, 2018) classifies cells by measuring their similarity to either the centroids of reference clusters (scmap-cluster) or by kNN cell annotation (scmap-cell). Finally, scPred (Alquicira-Hernandez *et al.*, 2019) reduces the reference data’s dimensionality using principal component analysis (PCA) and applies a support vector machine model for classification. The common unwanted characteristic of discriminative classification methods is that they do not utilize the distribution of genomic data. However, the distribution of genomic data carries key features of scRNA-seq data, which should be helpful for cell-type annotation. For example, ‘dropout’ is the well-known sparsity issue characterized by the excessive amount of zero counts in scRNA-seq data, arising from technical limitations in detecting moderate or low gene-expression levels in cells of the same type (Hicks *et al.*, 2018). Various imputation methods have been developed to remove dropouts from data, such as SAVER (Huang *et al.*, 2018), scImpute (Li and Li, 2018) and DrImpute (Gong *et al.*, 2018). However, imputation could generate false positive signals within the data due to the intrinsic circularity of current scRNA-seq expression recovery practices (Andrews and Hemberg, 2018). Furthermore, the proportion of dropouts can provide helpful information for cell annotation. To illustrate the importance of dropouts, we did two proportions *Z*-test on proportional zero of each gene between every pair of cell populations. In the selected dataset PBMC.10Xv2 (Ding *et al.*, 2019), we observed 10–48% of genes whose proportion of dropouts was significantly different in each pair of cell populations. The results details are given in Supplementary Table S1. These results suggest that genes with dropout carry important information helping distinguish cell types. Hence, instead of removing genes with dropout, we are motivated to build a novel model that fully utilizes the dropout information in the annotation.

To fully utilize the unique characteristics of scRNA-seq genomic data, we investigate the generative classification approach, which models the distribution of genomic data conditional on cell type. Such an approach focuses on distributions of genomic data in different cell types, and annotates cells using the Bayesian theorem. To the best of our knowledge, scID (Boufea *et al.*, 2020) is the only cell-type annotation method based on a generative classifier. scID uses Fisher’s linear discriminant analysis (LDA) to distinguish the characteristic genes of pre-determined cell clusters. LDA assumes that genomic data follow a multivariate normal distribution, which might over-simplify the complexity of the data. Furthermore, from data with limited sample sizes, it is hard to precisely estimate numerous parameters in the high-dimensional covariance matrix of the assumed multivariate normal distribution.

This article proposes a novel generative classifier for automated cell-type annotation, scAnnotate. We focus on addressing the two critical challenges of scRNA-seq data as discussed above: the curse of high dimensionality (as discussed in LDA) and explicitly modelling dropout. To address the curse of high dimensionality, we use every gene to make a classifier and consider it as a ‘weak’ learner, and then use a combiner function to ensemble ‘weak’ learners built from all genes into a single ‘strong’ learner for making the final decision. To select a gene’s distribution that explicitly models the excessive zero counts in each weak learner, we borrow the idea from differential expression (DE) analysis of scRNA-seq data. The literature on DE analysis is well-established, with many methods that focus on modelling excessive zero counts. For example, Kharchenko *et al.* (2014) introduced a Bayesian approach to scRNA-seq DE analysis in which non-zero counts are modelled using a Negative Binomial distribution and zero counts are modelled with a low-magnitude Poisson process. DEsingle (Miao *et al.*, 2018) is another scRNA-seq DE analysis tool that uses the Zero-Inflated Negative Binomial (ZINB) distribution. However, after batch effect removal and other preprocessing, scRNA-seq data are often no longer integers and hence are unsuitable for the ZINB model. Furthermore, recent benchmark studies did not show any clear advantage of the ZINB model in DE analysis of scRNA-seq data (Soneson and Robinson, 2018). We, therefore, model gene expression levels as a continuous variable. MAST (Finak *et al.*, 2015) joint models the proportion of dropouts and the distribution of non-dropouts using a hurdle regression model, which is one of the most popular DE analysis software, and has shown great performance in benchmark studies (Soneson and Robinson, 2018). Inspired by MAST, we joint model the proportion of dropouts and the gene expressions of non-dropouts by a two-component mixture model. We tried various distributions to model the non-dropout component in the mixture model and found empirically that the log-normal distribution works best for most of the data that we explored. In Section 4, we also discuss two alternative distributions implemented in our software that are useful in particular situations. In the rest of this article, we will introduce the details of the scAnnotate method and use real scRNA-seq datasets to compare its classification performance with nine other scRNA-seq annotation methods based on supervised machine learning algorithms.

## 2 Materials and methods

**Fig. 1.**
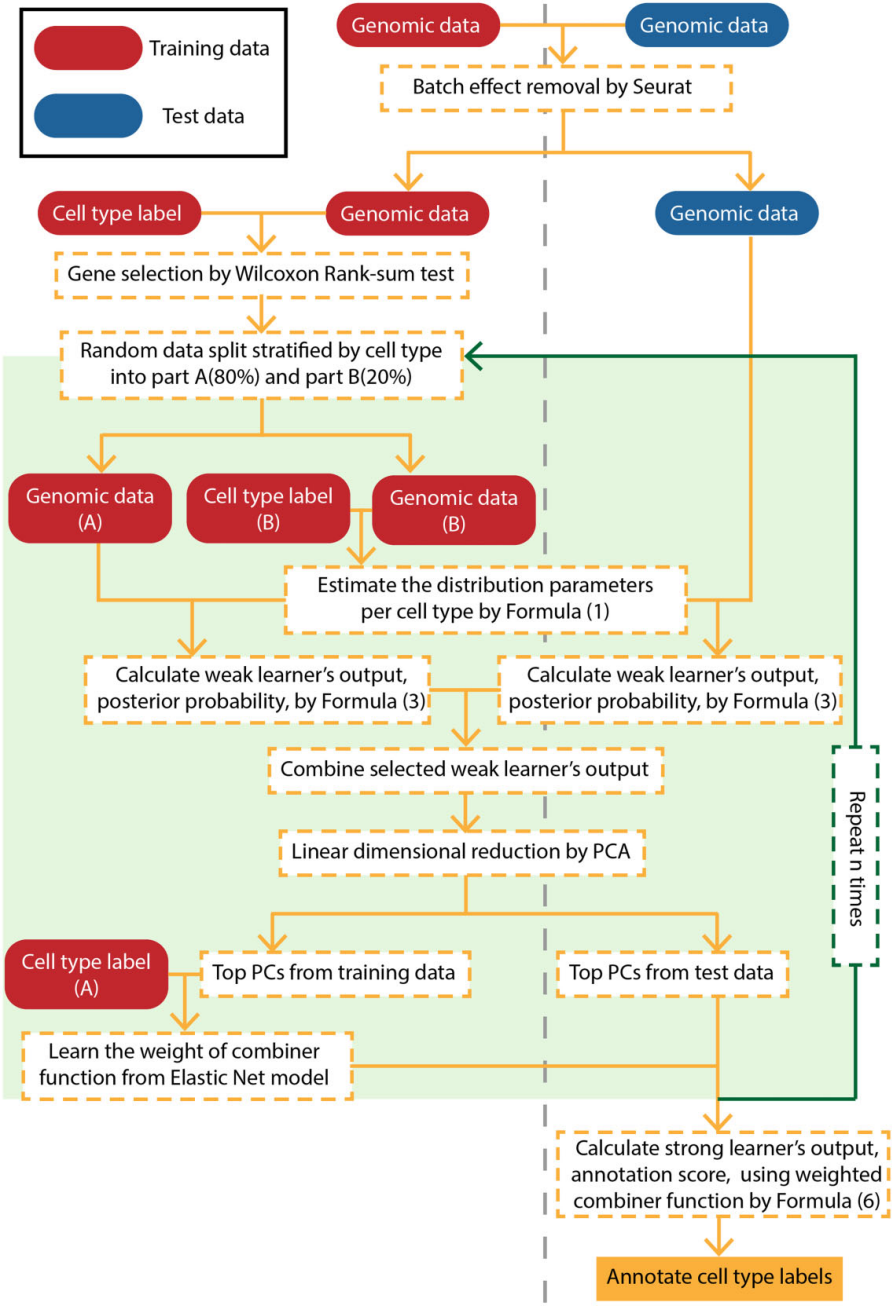
Workflow of scAnnotate on a dataset with at most one rare cell population (at most one cell population less than 100 cells). The vertical grey dashed line separates training data (left) and test data (right) information

**Fig. 2.**
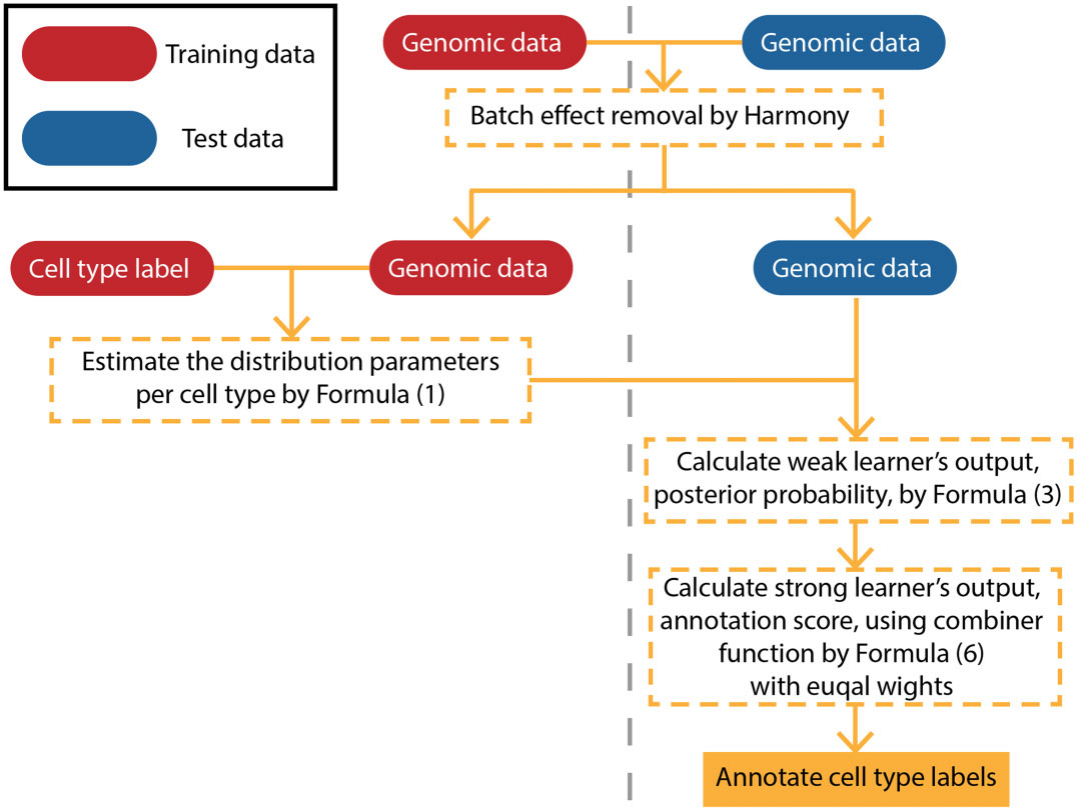
Workflow of scAnnotate on a dataset with at least two rare cell populations (at least two cell populations less than 100 cells). The vertical grey dashed line separates training data (left) and test data (right) information

We introduce scAnnotate, an automated cell-type annotation tool. scAnnotate is entirely data-driven, meaning that it requires training data to learn the classifier but does not require biological knowledge or subjective decisions from the user. It consists of three steps: preprocessing training and test data, model fitting on training data and cell classification on test data. The classification model in the last step uses an ensemble machine learning approach involving many weak learners and a combiner function to integrate the outputs of the weak learners into a single strong learner. Each weak learner is a classifier based on a mixture model for the expression level of one gene. The combiner is a weighted average of all weak learners’ outputs. The weights can be either learned from training data with at most one rare cell population or prespecified as equal weights on the training data with at least two rare cell populations. In this study, we defined a rare cell population as a cell population with less than 100 cells in a given dataset. scAnnotate handles data with varying numbers of rare cell populations differently. An illustration of its workflow is shown in Figure 1 (dataset with at most one rare cell population) and Figure 2 (dataset with at least two rare cell populations). Details of each element of scAnnotate will be discussed in the rest of this section.

### 2.1 Batch effect removal

When building a supervised machine learning model for cell-type annotation, batch effects often create differences between the training and testing data. We, therefore, believe that removing batch effects will make the model learned from the training data more suitable for annotating cells in the test data. For example, scPred (Alquicira-Hernandez *et al.*, 2019) has batch effect removal as a built-in optional step. Following this idea, we suggest using batch effect removal as a data preprocessing step unless users strongly believe that their training data and test data are similar enough to each other. scAnnotate removes batch effects using the Seurat package (Hao *et al.*, 2021) for data with at most one rare cell population or the Harmony package (Korsunsky *et al.*, 2018) for data with at least two rare cell populations. Both packages are recommended by Tran *et al.* (2020) due to their consistently high-quality performances and comparatively low runtimes. The output from the batch effect removal step is used as input for the classification model discussed next.

### 2.2 Mixture model for the expression level of a given gene in a fixed cell type

The traditional mixture model is a model-based clustering approach and does not need training data with cell-type labels. Our model has the same form as a mixture model, but we used the training data with cell-type labels to avoid the expectation–maximization (EM) algorithm for model fitting. Given the cell-type labels, we could estimate each gene’s density in each cell type directly without an iterative update. We use a mixture model to describe the expression level, $F_{ij}$, as below where $i=1,2,\ldots ,n_{t}$ is the index of cell types, $j=1,2,\ldots ,n_{g}$ is the index of (selected) genes, $F^{0}$ is the degenerated distribution at 0, $F_{ij}^{+}$ is a distribution supported on (0,∞) and $n_{t}$ and $n_{g}$ are, respectively, the total number of cell types and the total number of genes selected for use in classification. The $p_{ij}$ and $\left(1-p_{ij}\right)$ are the mixing proportions for $F^{0}$ and $F_{ij}^{+}$, respectively. Model (1) includes commonly used zero-inflated models such as the zero-inflated Poisson model as special cases, but it offers more flexibility than such zero-inflated models as it models the proportion of zeros and the distribution of the positive expression levels $F_{ij}^{+}$ separately. In particular, under Equation (1), all distributions supported on (0,∞) or a subset of (0,∞) may be used to model $F_{ij}^{+}$. In situations where no good parametric models for $F_{ij}^{+}$ are available, we may also specify $F_{ij}^{+}$ nonparametrically.


(1) $$F_{ij}=p_{ij}F^{0}+(1-p_{ij})F_{ij}^{+},$$

Note that the indexes *i* and *j* are used consistently throughout this article to refer to cell types and genes, respectively. To be concise, we will use these two indexes without repeated definitions in the rest of this article.

### 2.3 Mixture model for the expression level of a given gene, its estimation and prior specification

Let $\pi_{i}$ be the prior probability that a randomly selected cell is of type-*i* where $\pi_{1}+\pi_{2}+\cdots +\pi_{n_{t}}=1$. The (prior) distribution of the expression level of the *j*th gene of this cell is the following mixture distribution: where $F_{ij}$ are defined in Equation (1). We estimate $F_{ij}$ and $F_{j}$ using training data as follows. Suppose the training data contain $m_{i}$ independent type-*i* cells. Then, there are $m_{i}$ independent observations for the *j*th gene in type-*i* cells. Let $k_{ij}^{0}$ be the number of zeros among these $m_{i}$ observations and $k_{ij}^{+}$ be the number of positive observations so that $m_{i}=k_{ij}^{0}+k_{ij}^{+}$. Then, we may estimate the mixing proportions $p_{ij}$ in Equation (1) with


(2) $$F_{j}=\pi_{1}F_{1j}+\pi_{2}F_{2j}+\cdots +\pi_{n_{t}}F_{n_{t},j},$$

$${\hat{p}}_{ij}=k_{ij}^{0}/m_{i},\;i=1,2,\ldots ,n_{t},j=1,2,\ldots ,n_{g}.$$

To estimate the parameters of the distribution $F_{ij}^{+}$, we use the $k_{ij}^{+}$ positive observations. For example, if $F_{ij}^{+}$ is assumed to be a log-normal distribution, then we can find the maximum-likelihood estimates for its parameters using the $k_{ij}^{+}$ positive observations.

The prior probabilities $\pi_{i}$ depend on the application at hand. In the absence of information for determining these probabilities, we recommend the uniform prior $\pi_{i}=1/n_{t}$. We have also used the observed proportions $\pi_{i}=m_{i}/\sum_{k=1}^{n_{t}}n_{k}$, which are reasonable when the training sample is a random sample from the population of cells of all types.

### 2.4 Weak learner based on mixture model of a single gene

To classify a future cell of unknown type into one of the $n_{t}$ types with its $n_{g}$ gene expression data, we first use the $n_{g}$ genes one at a time to perform the classification. This leads to $n_{g}$ weak learners.

Let $X_{j}$ be the expression level of the *j*th gene of the cell. Then, since the cell type is unknown, $X_{j}∼F_{j}$ in Equation (2). Let $x_{j}$ be the observed value of $X_{j}$. The posterior probability that the cell belongs to type-*i* is which can be computed by using the estimated $F_{ij}$ and $F_{j}$. Specifically, for $x_{j}=0$, we have which is the probability that the observed zero comes from a type-*i* cell. For $x_{j}>0$, we have where ${\hat{f}}_{ij}$ is the estimated probability mass/density function of $F_{ij}^{+}$. When $F_{ij}^{+}$ is a continuous distribution, ${\hat{f}}_{ij}$ is a continuous density function, so $q_{ij}$ in Equation (4) is not a real probability, but we still call it a posterior probability here for the purpose of classifying the cell. If we only use the expression level of the *j*th gene, we would assign the cell to type-$i^{*}$ where by the rule of the maximum posterior probability. This is a weak learner in the sense that it is based on the expression level of only one gene.


(3) $$q_{ij}=P(\text{type}-i|X_{j}=x_{j})=\frac{P(X_{j}=x_{j}|\text{type}-i)P(\;\text{type}-i)}{P(X_{j}=x_{j})},$$

$$q_{ij}=P(\text{type}-i|X_{j}=0)=\frac{\pi_{i}{\hat{p}}_{ij}}{\pi_{1}{\hat{p}}_{1j}+\pi_{2}{\hat{p}}_{2j}+\cdots +\pi_{n_{t}}{\hat{p}}_{n_{t}j}},$$

(4) $$q_{ij}=\frac{\pi_{i}(1-{\hat{p}}_{ij}){\hat{f}}_{ij}(x_{j})}{\pi_{1}(1-{\hat{p}}_{1j}){\hat{f}}_{1j}(x_{j})+\cdots +\pi_{n_{t}}(1-{\hat{p}}_{n_{t}j}){\hat{f}}_{n_{t}j}(x_{j})},$$

(5) $$q_{i^{*}j}=\max\{q_{1j},q_{2j},\ldots ,q_{n_{t}j}\}$$

### 2.5 Combiner functions and the strong learner

Based on $n_{g}$ genes, we obtain $n_{g}$ weak learners to annotate cell types. Next, we define a combiner function to integrate the $n_{g}$ posterior probabilities into one final decision, which is considered a strong learner. We propose the combiner function as the weighted sum of log-transformed $q_{ij}$-scores, defined below, where the values of weights parameters $w_{j}$ are specified using two different approaches according to the sample size of the training data.


(6) $$s_{i}=s_{C}(q_{i1},q_{i2},\ldots ,q_{i,n_{g}})=\sum_{j=1}^{n_{g}}w_{j}\;\text{log}(q_{ij}),$$

Based on the scores obtained from the combiner function, we classify a cell to be the cell type with the largest score of $s_{i}$, that is, type-$i^{*}$ satisfying


(7) $$i^{*}=\underset{i}{\arg\max}\{s_{1},s_{2},\ldots ,s_{n_{t}}\}.$$

We call steps (1)–(7) mixture model-based supervised classification of a cell. We name this method ‘scAnnotate’.

When the training data have at most one rare cell population, we randomly split the training data into two parts. The weights $w_{j}$ are learned via the following five steps. (1) We use 20% of the cells to estimate the parameters of $F_{ij}$. (2) We use the estimated distributions to calculate $q_{ij}$ scores of the remaining 80% of cells. (3) Using the Wilcoxon rank-sum test, we filter out genes whose $q_{ij}$ scores are not highly associated with cell-type labels, that is, retain the top genes with the smallest *p*-values. (4) Using these 80% cells’ $q_{ij}$ scores as predictors and their corresponding cell types as outcomes, we train an Elastic Net model (Zou and Hastie, 2005) to learn the weights $w_{j}$. Note, to reduce the number of predictors, we apply PCA to the scores and use PC scores to replace the $q_{ij}$ scores. Since the Elastic Net model’s result is a linear combination of PC scores, and the PC scores are linear combinations of $q_{ij}$ scores, the final results are linear combinations of $q_{ij}$ scores. (5) To avoid sampling bias introduced by random data splitting, we repeat Steps (1)–(4) 10 times with different random splits, and use average weights learned from the 10 models as the final weights of combiner $s_{C}$.

When the training data have at least two rare cell populations, we do not have enough data to estimate parameters of all $F_{ij}$ and, at the same time, train a model to learn the weights $w_{j}$ for the combiner. Specifically, a rare cell population has less than 100 cells, as defined for this study. Since we only use 20% of training data to learn the distribution parameters, if we also have to model the weights $w_{j}$ for the combiner, we cannot sufficiently estimate the parameter $F_{ij}$ for a rare cell population with less than 20 cells. We can make a reasonable prediction when there is only one rare cell population. The well-estimated distribution of other cell populations can draw an excellent boundary to distinguish the only rare cell population from them. However, we cannot distinguish these rare cells from each other when there are at least two rare cells. In this case, we use all training cells to estimate the parameters of $F_{ij}$ and assume equal weights $w_{j}=1$ for all *j*. When we use a uniform prior $\pi_{i}=P(\text{type}-i)=1/n_{t}$ and equal weights $w_{j}=1$ with combiner $s_{C}$, it is equivalent to $\Pi_{j=1}^{n_{g}}q_{ij}\propto \prod_{j=1}^{n_{g}}P(X_{j}=x_{j}|\text{type}=i)$, and our ensemble learning reduces to the well-known Naive Bayes Classifier (Rish *et al.*, 2001). To see this, a detailed process of proven is given in Supplementary Section S1.

Note that we set 100 cells as the threshold for using our non-equal-weight ensemble model, which is an empirically ‘safe’ threshold to estimate the model parameters reasonably well. It implies using 20+ cells to estimate two distribution parameters (mean and SD of a log-normal distribution) and 80+ cells to train a model learning the best ensemble weights. Users could set a smaller threshold and still get good annotation results. However, the threshold value must not be too small. When the sample size is insufficient to reasonably estimate the model parameters, the equal-weights ensemble model may work better since assuming equal weights enables using all cells to estimate distribution parameters. Note that other combiner functions could be used as alternatives to Formula (6). We provided a few examples in Supplementary Section S2. We investigated the combiners using multiple real scRNA-seq datasets and empirically found the combiner $s_{C}$ works best.

### 2.6 Implementation of scAnnotate

Classification using scAnnotate depends on three key components: (i) the model for $F_{ij}^{+}$ in Equation (1), (ii) the prior probabilities $\pi_{i}$ in Equation (2) and (iii) the combiner function in Equation (6). For (i), we may use, for example, Negative Binomial distribution, Exponential distribution, log-normal distribution or in situations where no good parametric models for positive expression levels are available, a nonparametric measure (see Section 2.7). Due to the usually huge number of combinations of *i* (cell types) and *j* (genes), it is not practical to model each individual $F_{ij}^{+}$ separately, so we assume that distributions of all $F_{ij}^{+}$ are of the same type, for example, all log-normal; and they can only differ in their parameter values. For (ii), we use either a uniform prior or the observed proportions as discussed in Section 2.3. For (iii), we may use one of the three combiner functions given above.

In real applications, we recommend using several combinations of the three components to build several classifiers with the training data, and then use test data to evaluate the performance of these classifiers using their $F_{1}$ scores to identify the optimal combination with the highest $F_{1}$ score. For real data, the correct specifications of the components are unknown, so optimizing the combination by trying several combinations and choosing the best one protects scAnnotate from serious misspecifications of its components. For examples that we have tried, we found that the combination of the log-normal distribution, uniform prior and combiner $s_{C}$ often has the best or second-best performance. For simplicity of presentation, this combination is used in all examples in the next section.

Note that in cross-species and cross-platform studies, we need to apply batch effect removal techniques to preprocess the datasets and the processed datasets contain no zeros. For such processed datasets, the mixture model $F_{ij}$ in Equation (1) for the *j*th gene of a type-*i* cell reduces to $F_{ij}^{+}$ as the proportion of zero $p_{ij}=0$. The mixture model $F_{j}$ in Equation (2) remains unchanged. The implementation of scAnnotate also remains the same.

### 2.7 Nonparametric depth measure

The true distribution of gene expression can be very complex. We may be unable to find, from the set of commonly used parametric distributions, a suitable one for modelling $F_{ij}^{+}$. The assumption that distributions for $F_{ij}^{+}$ of all genes are the same kind and that they can only differ in parameter values may also be too strong. To deal with these issues, when the sample size is large, we suggest using a nonparametric depth measure for $F_{ij}^{+}$, which is totally free of any parametric assumptions. Specifically, we may replace the estimated density function ${\hat{f}}_{ij}$ with a depth measure when computing the posterior probability in Equation (4). We now illustrate this point using the halfspace depth measure (Liu *et al.*, 1999).

For a fixed gene of a fixed cell type, suppose there are *m* non-zero expression data points from the training dataset $x_{1},x_{2},\ldots ,x_{m}$. Let $x^{*}$ be the expression level of that gene of the cell to be classified. The halfspace depth of $x^{*}$ measures how consistent $x^{*}$ is with the sample $x_{1},x_{2},\ldots ,x_{m}$. It is defined as follows. First, rank the m+1 observations in the augmented sample $x_{1},x_{2},\ldots ,x_{m},x^{*}$ and denote by $r(x^{*})$ the rank of $x^{*}$. Then, the halfspace depth of $x^{*}$, $h(x^{*})$, is given by


$$h(x^{*})=\frac{1}{m+1}\min\{r(x^{*}),(m+1)-r(x^{*})+1\}.$$

To see how $h(x^{*})$ measures the consistency of $x^{*}$ with the data, when $x^{*}$ is the smallest or the largest of the augmented sample, $h(x^{*})$ has its minimum value of 1/(m+1), so a low $h(x^{*})$ value indicates $x^{*}$ is not consistent with the data in that it is an extreme value. On the other hand, when $x^{*}$ is the median of the augmented sample, $r(x^{*})=(m+1)/2$ and $h(x^{*})$ reaches its maximum value of 1/2, so a large $h(x^{*})$ value (close to 1/2) indicate $x^{*}$ is consistent with the data. As such, it may be used to substitute ${\hat{f}}_{ij}$ in scAnnotate. Using this depth measure protects scAnnotate from severe misspecification of the model for $F_{ij}^{+}$. In simulation studies, scAnnotate based on the halfspace depth outperforms scAnnotate with severely misspecified $F_{ij}^{+}$ in terms of accuracy. On the other hand, the depth measure requires the number of non-zero observations at all genes to be large (otherwise, the depth measure is too discrete to be a useful replacement for ${\hat{f}}_{ij}$) and is more computationally intensive.

To evaluate the performance of scAnnotate, we conduct a benchmark study to compare it against nine other scRNA-seq annotation methods based on supervised machine learning algorithms, including scID (Boufea *et al.*, 2020), scClassify (Lin *et al.*, 2020), SingleCellNet (Tan and Cahan, 2019), scPred (Alquicira-Hernandez *et al.*, 2019), CaSTLe (Lieberman *et al.*, 2018), SingleR (Aran *et al.*, 2019), CHETAH (de Kanter *et al.*, 2019), scmapCluster and scmapCell (Kiselev *et al.*, 2018). The parameters of these methods are chosen according to the suggestions in their vignettes or the software default settings; we note that all of the benchmarked methods have a fully automated data-driven approach without requiring previous biological knowledge. Details of the nine methods are listed in Supplementary Table S2. In our experiments, all models are learned on training data and then applied to annotate cells in the test data. Prior to performing classification, we remove all cells whose cell types do not appear in both the training and test data. To evaluate the classification performance of the benchmarked methods, we compare the predicted labels of the test data with the corresponding true labels. Following the evaluation rule of other annotation method papers (Alquicira-Hernandez *et al.*, 2019; Lieberman *et al.*, 2018; Lin *et al.*, 2020; Zhao *et al.*, 2020), we use classification accuracy as our performance criteria. Accuracy in this study is defined as the percentage of correctly annotated cells.

We conduct our benchmark study under three situations according to the relationship between training and test data: (1) training and test data are from the same platform (i.e. obtained from the same sequencing method) and the same species; (2) training and test data are from different platforms and (3) training and test data are from different species, human versus mouse.

### 2.8 Datasets and preprocessing

Supplementary Table S3 summarizes the 14 publicly available scRNA-seq datasets used in our benchmark study. These data have been used to illustrate the annotation performances of scAnnotate and its nine competitor methods in this section.

The Seurat (version 4.0.5) (Hao *et al.*, 2021) package was used for normalization on all raw count matrices. The datasets were normalized using the NormalizeData function with the ‘LogNormalize’ method and a scale factor of 10 000. Since scID was not compatible with log-transformed data, it used the raw count matrices as input. All other methods used the log-transformed normalized gene expression matrix as input.

## 3 Results

### 3.1 Annotation performance evaluation when training and test data are generated from the same species using the same platform

We used all 14 datasets as described in Supplementary Table S3 for intra-dataset evaluation. We applied stratified sampling (by cell type) to select 80% of the dataset as training data and set the remaining 20% of the dataset as test data. To eliminate the variability in performance evaluation caused by sampling bias, we repeated this experiment 10 times with different random data splitting. Then we reported the median accuracy from the 10 replications to represent each method’s overall performance on selected datasets.

**Fig. 3.**
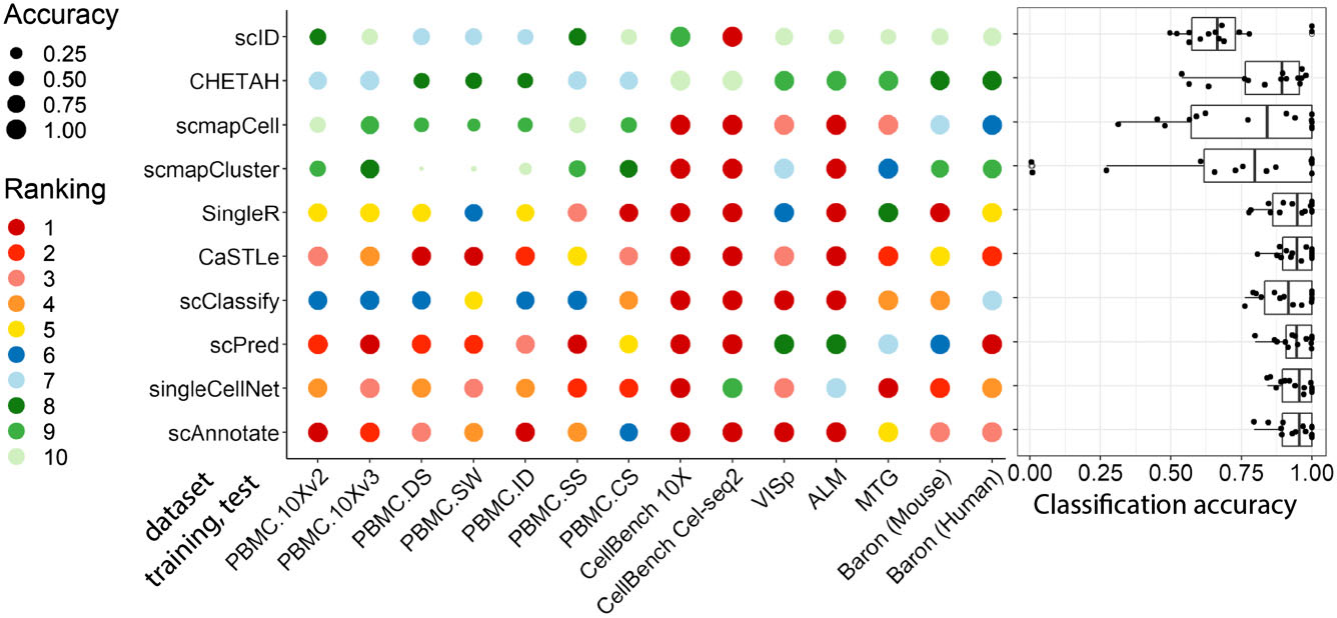
Within-study classification performance of scAnnotate on all 14 datasets, as provided by Ding *et al.* (2019), Tian *et al.* (2019), Baron *et al.* (2016), Tasic *et al.* (2018) and Hodge *et al.* (2019). The dot plot shows the comparison results of each individual setting. Each column represents one combination of training and test data. Each circle represents the performance of one method. The colours of the circles represent the methods’ ranks and the sizes of the circles represent their corresponding accuracies. The boxplot on the right summarizes the overall comparison of methods under all settings

Figure 3 shows the classification accuracy of scAnnotate and competitor methods. scAnnotate (assuming $F_{ij}^{+}$ follows a log-normal distribution) had a mean accuracy 93.83%, a median accuracy 95.47%, and an overall prediction accuracy range of 79.43–100.00%. In most cases, scAnnotate ranks in the top four best-performing methods. Multiple methods had high classification accuracies when training and test data were similar.

### 3.2 Annotation performance evaluation using cross-platform training data

We used the PBMC datasets (Ding *et al.*, 2019) provided by the SeuratData Package and the lung cancer cell lines dataset (Tian *et al.*, 2019) to evaluate the 10 annotation methods’ classification performances when training and test data are obtained from different scRNA-seq generating platforms. The PBMC data consist of scRNA-seq data generated using seven different platforms as listed in Supplementary Table S3, which leads to 42 pairs of cross-platform training and test data. The lung cancer cell lines datasets, 10X and Cel-seq2, provided two pairs of cross-platform training and test data.

**Fig. 4.**
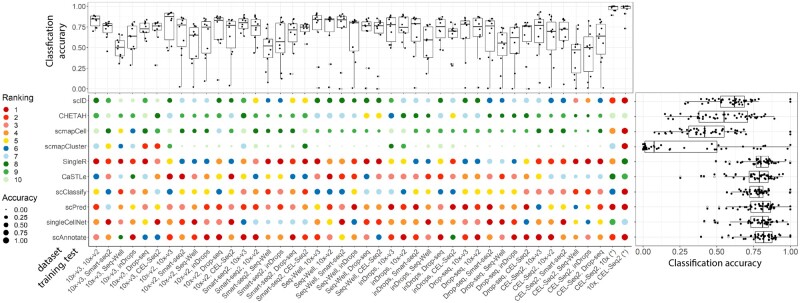
Classification performance of scAnnotate on 44 combinations of cross-platform datasets, as provided by Ding *et al.* (2019) and Tian *et al.* (2019), which included 42 PBMC datasets and two lung cancer cell lines datasets marked with (*). The dot plot shows the comparison results of each individual setting. Each column represents one set of training and test data combination. Each circle represents the performance of one method. The colours of the circles represent the methods’ ranks and the sizes of the circles represent their corresponding accuracies. The boxplot on the right side of the dot plot summarizes the overall comparison of methods under all settings. The boxplot on top of the dot plot summarizes the overall performance of classifiers for each experiment

scAnnotate (assuming $F_{ij}^{+}$ follows a log-normal distribution) had the highest mean accuracy (80.27%) and the highest median accuracy (81.92%), and an overall prediction accuracy range of 39.45–100.00%. scAnnotate, singleCellNet and scPred had the highest median accuracies, indicating that they are the top three best methods in the overall comparison of the 44 settings. When we look into individual settings, scAnnotate is among the top-ranked most accurate methods in most of the 44 cross-platform settings (Figure 4). When scAnnotate is not the best method, its accuracy is often not much lower than that of the winners. We note that there is a significant drop in performance for scAnnotate when trained on the PBMC 10x-v3 dataset and tested on the PBMC Seq-Well dataset. As shown in the top boxplot of Figure 4, all methods have a significant drop in performance for this cross-platform dataset combination; thus, in general, training on the 10x-v3 dataset may produce poor predictions on the Seq-Well dataset. Comparing the performance of each classifier across the different protocols on the lung cancer cell lines, we observe an almost perfect performance for all classifiers. To illustrate the difference between the top-performing methods, we did a paired Wilcoxon rank-sum test on scAnnotate and other top-performing methods. Based on 0.05 *p*-value criteria, we didn’t observe a significantly different performance between the scAnnotate and the other top four best-performing methods (singleCellNet, scPred and SingleR).

### 3.3 Annotation performance evaluation using cross-species training data

We first trained scAnnotate (assuming that $F_{ij}^{+}$ follows a log-normal distribution) and nine other cell annotation methods on the Baron *et al.*’s (2016) mouse pancreatic dataset and predicted 10 pancreatic cell types in the Baron *et al.*’s (2016) human pancreatic datasets. scAnnotate ranked first with a prediction accuracy of 92.23%. We then switched the training–testing order and used the human data as training data to classify the mouse data. scAnnotate’s rank is third, with a prediction accuracy of 90.15%.

**Fig. 5.**
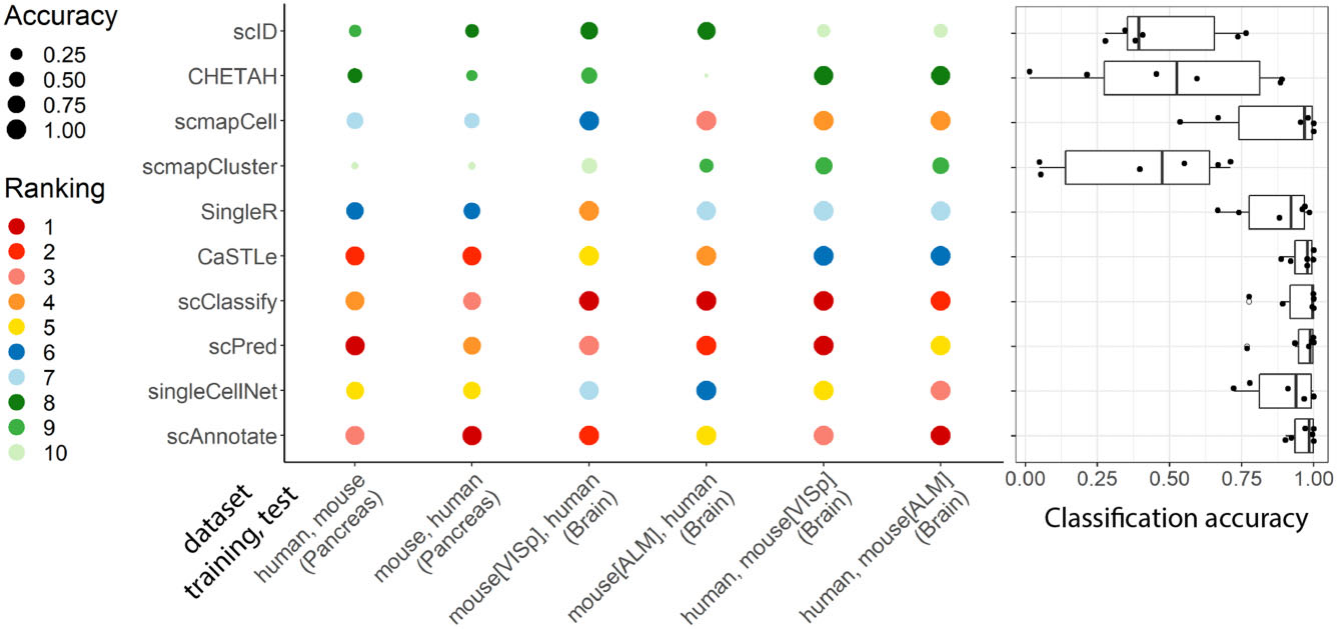
Cross-species classification performance of scAnnotate and nine other methods on six combinations of mouse and human scRNA-seq datasets, provided by Baron *et al.* (2016), Tasic *et al.* (2018) and Hodge *et al.* (2019). The dot plot shows the comparison results of each individual setting. Each column represents one combination of training and test data. Each circle represents the performance of one method. The colours of the circles represent the methods’ ranks and the sizes of the circles represent their corresponding accuracies. The boxplot on the right summarizes the overall comparison of methods under all settings

We then repeated this training/testing process with scAnnotate and the nine competitor methods on the mouse and human brain datasets provided by Tasic *et al.* (2018) and Hodge *et al.* (2019), respectively. Since we utilized two mouse brain datasets and one human brain dataset, switching the training-testing order gave us four distinct pairs of datasets. As shown in Figure 5, scAnnotate consistently ranks in the top five best-performing methods for mouse and human brain cell annotation with a mean accuracy of 99.15% and a median accuracy of 99.79%.

### 3.4 scAnnotate can complement other annotation methods

The scAnnotate uses a very distinct modelling approach compared with its competitors. It is the only annotation method that explicitly models the dropout (a critical characteristic of scRNA-seq), and is one of the only two generative classifiers while all others are discriminative classifiers. Therefore, we expect scAnnotate’s annotation of individual cells can be quite different from its competitors, although scAnnotate has similar cohort-level accuracy to other top-performing methods. That is, scAnnotate’s incorrectly annotated cells could be very distinct from its competitors’ incorrectly annotated cells. Being able to complement competitors enables scAnnotate to be used together with other annotation methods, which may lead to improved results. The mosaic plot shows the cells that are inconsistently annotated by the top six benchmarked methods in 42 PBMC cross-platform datasets given in Supplementary Figures S1–S3.

**Fig. 6.**
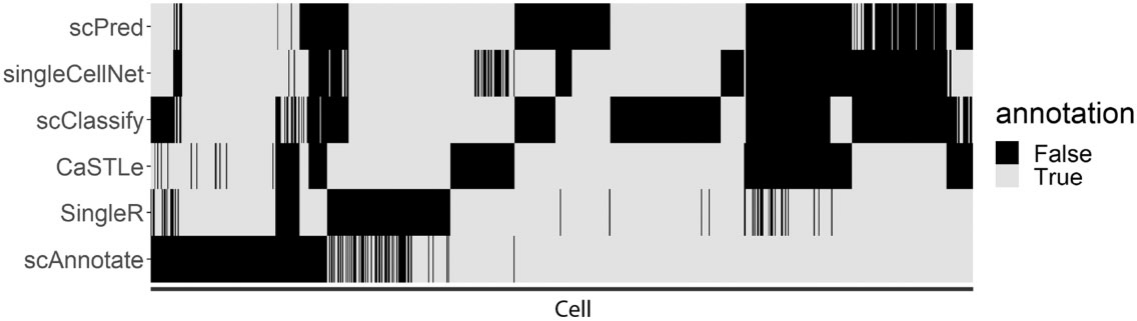
The mosaic plot shows the cells of the PBMC.10Xv3 dataset that are inconsistently annotated by the top six benchmarked methods (when trained on the PBMC.SW dataset)

Next, we look into the detailed annotation results of one data analysis conducted above, as an example, to demonstrate the difference between annotation results of scAnnotate’s generative approach and its competitors (i.e. discriminative methods). We focus on the cross-platform analysis training model on PBMC.SW dataset and tested it on the PBMC.10Xv3 dataset (see Supplementary Table S3 for dataset details). Of the 2930 cells included in the PBMC.10Xv3 dataset, 886 cells were inconsistently annotated by these methods. The mosaic plot of Figure 6 shows the comparison of annotation results of six top-performing annotation methods. Each row represents an annotation method and each column represents a cell. The grids filled with black colour indicate those cells are incorrectly annotated. To highlight the difference pattern between methods’ annotation results, we only show the results of the top six best-performing methods and exclude cells consistently annotated by all methods. We grouped these cells into 20 clusters using the *K*-means algorithm, and re-ordered them according to their cluster membership to highlight the pattern. From Figure 6, we can observe an obvious pattern that scAnnotate’s results complement all other methods. In this analysis, SingleR had the best performance with an accuracy of 91.74% and scAnnotate ranked second with an accuracy of 90.14%. If we could let methods correct each other’s errors (e.g. by manually investigating inconsistently annotated cells or building an ensemble model to borrow information among different methods), the ideal accuracy could be improved to 98.50% in this example.

## 4 Discussion

scAnnotate is an analysis framework that consists of three major components. Users can change the components according to their own needs. First, we use two available software as a batch effect removal step: Harmony for datasets with at least two rare cell populations and Seurat for datasets with at most one rare cell population. Many batch effect removal methods can be used to replace them, such as the methods compared in the benchmark study by Tran *et al.* (2020). Note that the batch effect removal step is critical, which ensures that training and test data are comparable so the model learned on the training data can be applied to test data. This step requires learning a new annotation model for every test data, which is ‘not’ desired for conventional classification problems, but is essential and practical in cell annotation problems. In practice, when users want to annotate cells from scRNA-seq data, they should find the best matching training data preferably generated from the same species using the same platform. Using pre-trained models as a black box for annotation could lead to misleading results. Second, an Elastic Net (Zou and Hastie, 2005) model is used to learn a combiner function. Users can choose any supervised machine learning model to replace it. For example, when the sample size is large, users can train the combiner function using XGBoost (Chen and Guestrin, 2016), which is faster and much more precise than Elastic Net in this situation. Note that the supervised learning approach can only annotate cell types seen in training data. Semi-supervised learning methods could be incorporated into this framework as follow-up research to annotate unseen cell types. Third, the distributions used in weak learners can also be changed. We will discuss this next.

After investigating many candidate distributions used in weak learners, we recommend using the log-normal distribution as the non-dropout component of our mixture model. Besides log-normal, we added alternative distributions in our software for particular usages. The non-parametric distribution is estimated using a depth function approach for extra-large sample size problems. We believe that all distributions oversimplify the complex truth of gene expression. Hence, a non-parametric approach can better estimate the complex true distribution when the sample size is big enough. We believe that this alternative will be more useful in the future as the sample size of scRNA-seq data continues to grow rapidly over the years.

DE analysis uses genes one at a time; most DE methods, therefore, focus on modelling gene expression distribution to best utilize key features of genomic data. In contrast, annotation analysis needs to use all genes to make decisions. Most cell annotation methods do not model the distribution of genomic data, since it is hard to model the joint distribution of many genes. We address this challenge using an ensemble approach. We build classifiers using each gene separately and ensemble them using a combiner. This approach has three advantages in computing. First, by modelling each gene separately, the number of parameters in our model is linear in the number of genes, which successfully avoids the curse of dimensionality. Second, the estimation of distribution parameters in the training data and the evaluation of posterior probability in the test data often have close-formed formulas, rather than the expensive iterative approximation used by many other types of classifiers. Third, when necessary, such calculations for tens of thousands of genes can be done in parallel to further reduce computing time.

While scAnnotate is developed as a standalone tool for cell annotation, it can also be used with other methods to correct each other’s errors. According to the ‘No free lunch theorem’ by Wolpert and Macready (1997), there is no single best machine learning algorithm for predictive modelling problems. All methods have their particular advantages and disadvantages. Hence, different annotation methods are expected to make errors on different sets of cells. At the end of Section 3, we demonstrate that scAnnotate’s annotation errors are very different from its competitors. In practice, users could use scAnnotate and another method to analyse the same data and compare their annotation results. Users could manually investigate the cells annotated inconsistently between scAnnotate and its competitors to improve the accuracy of cell annotation further. When there are enough computing resources and training data, users could also build an ensemble annotator to integrate the annotation results of many methods automatically. In such an ensemble annotator, we believe scAnnotate should play a key role because of its distinct characteristics and ability to complement competitors. The ensemble annotator is not limited to the competing methods selected in our study. There were many new methods developed for cell-type annotation while we were developing the scAnnotate. scNym (Kimmel and Kelley, 2021) involved semi-supervised adversarial neural networks for classification. SciBet (Li *et al.*, 2020) was developed based on a multinomial-distribution model and maximum-likelihood estimation. Cell-ID (Cortal *et al.*, 2021) is based on multiple correspondence analysis. scSorter (Guo *et al.*, 2021) is a semi-supervised method that assigns cells to known cell types according to marker genes. scBERT (Yang *et al.*, 2022) is a large-scale pretrained deep language model for cell-type annotation. Users could also use annotation results of scAnnotate and other methods as input for downstream analyses, then compare the final results to identify which one makes more sense in biology.

In conclusion, we introduce scAnnotate, a streamlined process for scRNA-seq data analysis that includes data preprocessing and cell annotation. We simultaneously model genes’ dropout proportions and expression levels via a two-component mixture model. We use an ensemble machine learning approach to address the curse of high dimensionality. We build weak classifiers using each gene and use a combiner function to integrate all weak classifiers into a single strong classifier. Using multiple real scRNA-seq benchmark datasets, we show that scAnnotate accurately annotates cells when training and test data are from (1) the same platform and species, (2) different scRNA-seq generating platforms and (3) different species (specifically, mouse to human). Compared with other supervised machine learning methods, scAnnotate provides top-tier cell classification performance.

## Supplementary Material

vbad030_Supplementary_Data
