# Supplementary material for: scAnnotate: an automated cell-type annotation tool for single-cell RNA-sequencing data
Source: Bioinform Adv. 2023 Mar 13;3(1):vbad030. doi: 10.1093/bioadv/vbad030 (PMC10027414; doi:10.1093/bioadv/vbad030)
Supplement: vbad030_Supplementary_Data [file vbad030_supplementary_data.pdf]

SUPPLEMENTARY MATERIAL

XIANGLING JI [1,], DANIELLE TSAO [1], KAILUN BAI [1], MIN TSAO [1], LI XING [2,*] AND XUEKUI ZHANG [1,*]

1. Using Naive Bayes Classifier as the Combiner Function

The weighted sum of log-transformed q_{ij}-scores are defined as

$$s_i = s_C(q_{i1}, q_{i2}, \ldots, q_{i,n_g}) = \sum_{j=1}^{n_g} w_j log(q_{ij}), \quad i = 1, 2, \ldots, n_t,\ j = 1, 2, \ldots, n_g,$$

When sample size is too small to estimate the weights w_j's, we use uniform prior $\pi_i = P(\text{type-}i) = 1/n_t$ and equal weights $w_j = 1$ in combiner s_C, which is equivalent to $\Pi_{j=1}^{n_g} q_{ij} \propto \prod_{j=1}^{n_g} P(X_j = x_j|\text{type} = i)$. In this case, our ensemble learning reduces to the Naive Bayes Classifier [1] (proof provided below), which is well-known for its great classification performance using high-dimensional and small sample size data.

The Naive Bayes Classifier uses posterior probability,

$$\begin{aligned} s_i^{nb} =& P(\text{type} = i|X_1, \ldots, X_{n_g}) \\ =& \frac{P(X_1, \ldots, X_{n_g}|\text{type} = i)P(\text{type} = i)}{P(X_1, \ldots, X_{n_g})} \\ =& \frac{\prod_{j=1}^{n_g} P(X_j = x_j|\text{type} = i)P(\text{type} = i)}{P(X_1, \ldots, X_{n_g})} \\ \propto& \prod_{j=1}^{n_g} P(X_j = x_j|\text{type} = i) = \Pi_{j=1}^{n_g} q_{ij} \\ =& exp\left(\sum_{j=1}^{n_g} log(q_{ij})\right). \end{aligned}$$

So, the Naive Bayes Classifier is a special case of s_C with a uniform prior and equal weights.

2. Other Examples Of Combiner Functions

We have several other examples of combiner function. The first example is the voting score combiner function

$$s_i = s_A(q_{i1}, q_{i2}, \ldots, q_{i,n_g}) = \sum_{j=1}^{n_g} I(q_{ij})$$

where $I(q_{ij}) = 1$ if $q_{ij} = \max\{q_{1j}, q_{2j}, \ldots, q_{n_tj}\}$ and $I(q_{ij}) = 0$ otherwise. This combiner function essentially counts the number of genes s_i that give the cell a type-i classification by the rule of maximum posterior probability shown in

$$q_{i^*j} = \max\{q_{1j}, q_{2j}, \ldots, q_{n_tj}\} \tag{1}$$

With this combiner function, scAnnotate classifies a cell as a type-i cell if it is most frequently classified/voted as a type-i cell by the n_g weak learners.

Another example is the weighted average of the posterior probabilities for type-i

$$s_i = s_B(q_{i1}, q_{i2}, \ldots, q_{i,n_g}) = \sum_{j=1}^{n_g} w_j q_{ij}$$

where the weight $w_j \geq 0$ and it represents the importance of the jth gene. Such importance may, for example, be a quantitative measure of how accurate the classification is when only $q_{1j}, q_{2j}, \ldots, q_{n_tj}$ are used to classify cells in the test data through (1). We may also apply the weights to define a weighted version of the voting score combiner,

$$s_i' = s_A'(q_{i1}, q_{i2}, \ldots, q_{i,n_g}) = \sum_{j=1}^{n_g} w_j I(q_{ij}).$$

3. **Supplementary Tables**

compared cell population	B cell	CD14+ monocyte	CD16+ monocyte	CD4+ T cell	Cytotoxic T cell	Dendritic cell	Megakar-yocyte	Natural killer cell	Plasmacytoid dendritic cell
B cell	-	8442	4858	5240	6503	9015	10421	4700	5100
CD14+ monocyte	-	-	5900	8806	7952	10731	9129	6948	7052
CD16+ monocyte	-	-	-	5007	5443	5655	9698	4106	3100
CD4+ T cell	-	-	-	-	5069	9339	10393	3652	5166
Cytotoxic T cell	-	-	-	-	-	10117	9865	2983	6174
Dendritic cell	-	-	-	-	-	-	11922	8523	2490
Megakaryocyte	-	-	-	-	-	-	-	9897	9711
Natural killer cell	-	-	-	-	-	-	-	-	4657
Plasmacytoid dendritic cell	-	-	-	-	-	-	-	-	-
This dataset contains 24671 genes, excluding the gene with all zero expression levels.									

Table S1. The number of genes that proportional zero are significantly different in the two cell types based on a two proportions Z-test with a p-value less than 0.05 criteria on the PBMC.10Xv2 dataset[2].

**Name**	**Version**	**Underlying classifier**	**Source**	**Reference**
scID	2.2	LDA	https://github.com/BatadaLab/scID	[3]
scClassify	1.5.1	Weighted kNN classifier	https://github.com/SydneyBioX/scClassify	[4]
SingleCellNet	0.1.0	Random Forest	https://github.com/pcahan1/singleCellNet/	[5]
scPred	1.9.2	SVM	https://github.com/powellgenomicslab/scPred	[6]
CaSTLe	GitHub:b43580b	XGBoost classifier	https://github.com/yuvallb/CaSTLe	[7]
SingleR	1.8.0	Correlation to training set	https://github.com/dviraran/SingleR	[8]
CHETAH	1.9.0	Correlation to training set	https://github.com/jdekanter/CHETAH	[9]
scmapCluster	1.16.0	Nearest median classifier	https://github.com/hemberg-lab/scmap	[10]
scmapCell	1.16.0	kNN	https://github.com/hemberg-lab/scmap	[10]

Table S2. The cell type annotation methods compared with our method in this study

Dataset	Description	Protocol	Cells	Genes	Cell types	Reference
PBMC.10Xv2	PBMC	10X Chromium (v2)	9806	33694	9	[2]
PBMC.10Xv3	PBMC	10X Chromium (v3)	3222	33694	8	[2]
PBMC.DS	PBMC	Drop-seq	6584	33694	9	[2]
PBMC.SW	PBMC	Seq-Well	3727	33694	7	[2]
PBMC.ID	PBMC	inDrops	6584	33694	9	[2]
PBMC.SS	PBMC	Smart-seq2	526	33694	7	[2]
PBMC.CS	PBMC	Cel-seq2	526	33694	7	[2]
CellBench 10X	Human lung cancer cell lines	10X chromium	3803	11778	5	[11]
CellBench Cel-seq2	Human lung cancer cell lines	Cel-seq2	570	12627	5	[11]
VISp	Mouse primary visual cortex	Smart-seq v4	12832	42625	3	[12]
ALM	Mouse anterior lateral motor area	Smart-seq v4	8758	42461	3	[12]
MTG	Human middle temporal gyrus	Smart-seq v4	14696	16161	3	[13]
Baron (Mouse)	Mouse pancreas	inDrops	1886	14878	13	[14]
Baron (Human)	Human pancreas	inDrops	8569	20125	13	[14]

Table S3. Datasets used in this study

The human Peripheral Blood Mononuclear Cells (PBMC) scRNA-seq data collection was downloaded from the SeuratData package with dataset name "pbmcsca" [2] and consists of seven datasets that were sequenced using seven different methods: 10x Chromium (v2), 10x Chromium (v3), Drop-seq, Seq-Well, inDrops, Smart-seq2, and Cel-seq2. For PBMC cell annotation, we removed all cells labelled as "Unassigned." Each dataset was then used as training data and all other datasets as test data. This gave us $7 * 6 = 42$ distinct pairs of cross-platform datasets. The human lung cancer cell lines data were downloaded from the Zenodo page provided by [15]. The CellBench 10X dataset was obtained from GSM3618014, and the CellBench Cel-Seq2 dataset was obtained from GSEM3618022, GSM3618023, and GSM3618024. We used both the CellBench 10X dataset and the CellBench Cel-seq2 dataset once as training data and once as test data. This gave us two more distinct pairs of cross-platform datasets.

The mouse and human pancreatic scRNA-seq data were downloaded from the National Center for Biotechnology Information (NCBI) Gene Expression Omnibus (GEO) for GSE84133 [14]. In preparation for cross-species cell annotation, we converted the human gene symbols to mouse ortholog gene symbols using the ortholog table provided by SingleCellNet [5]. The mouse and human brain datasets were downloaded from the Zenodo page by [15]. The analysis was limited to common genes between the training and test data. We first used the mouse data as training data and the human data as test data. We then switched the training-testing order and used the human data as training data in order to classify the mouse data. This gave us $1 * 2 + 2 * 2 = 6$ distinct pairs of cross-species datasets.

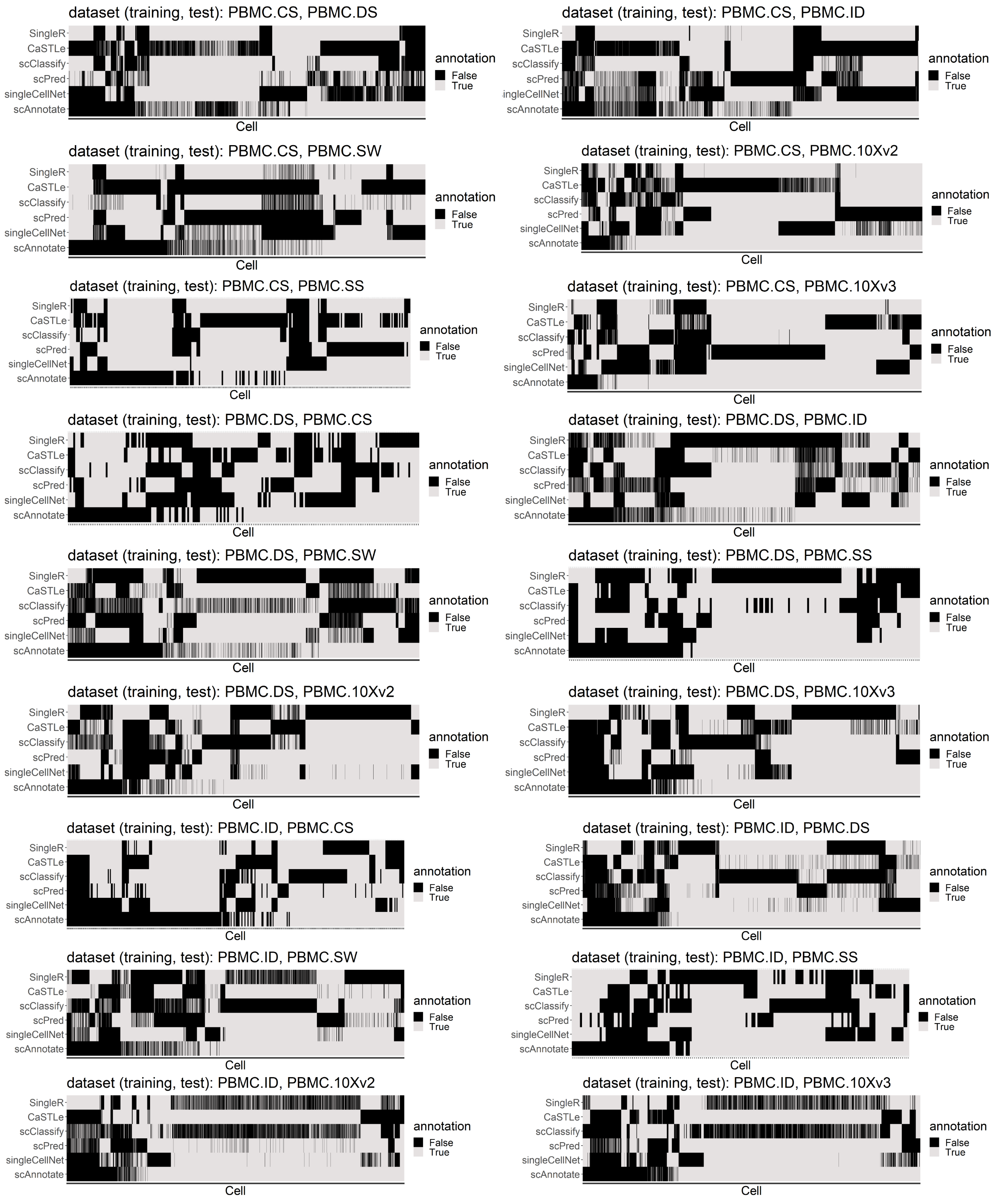

Figure S1. The mosaic plot shows the cells of each dataset that are inconsistently annotated by the top six benchmarked methods.

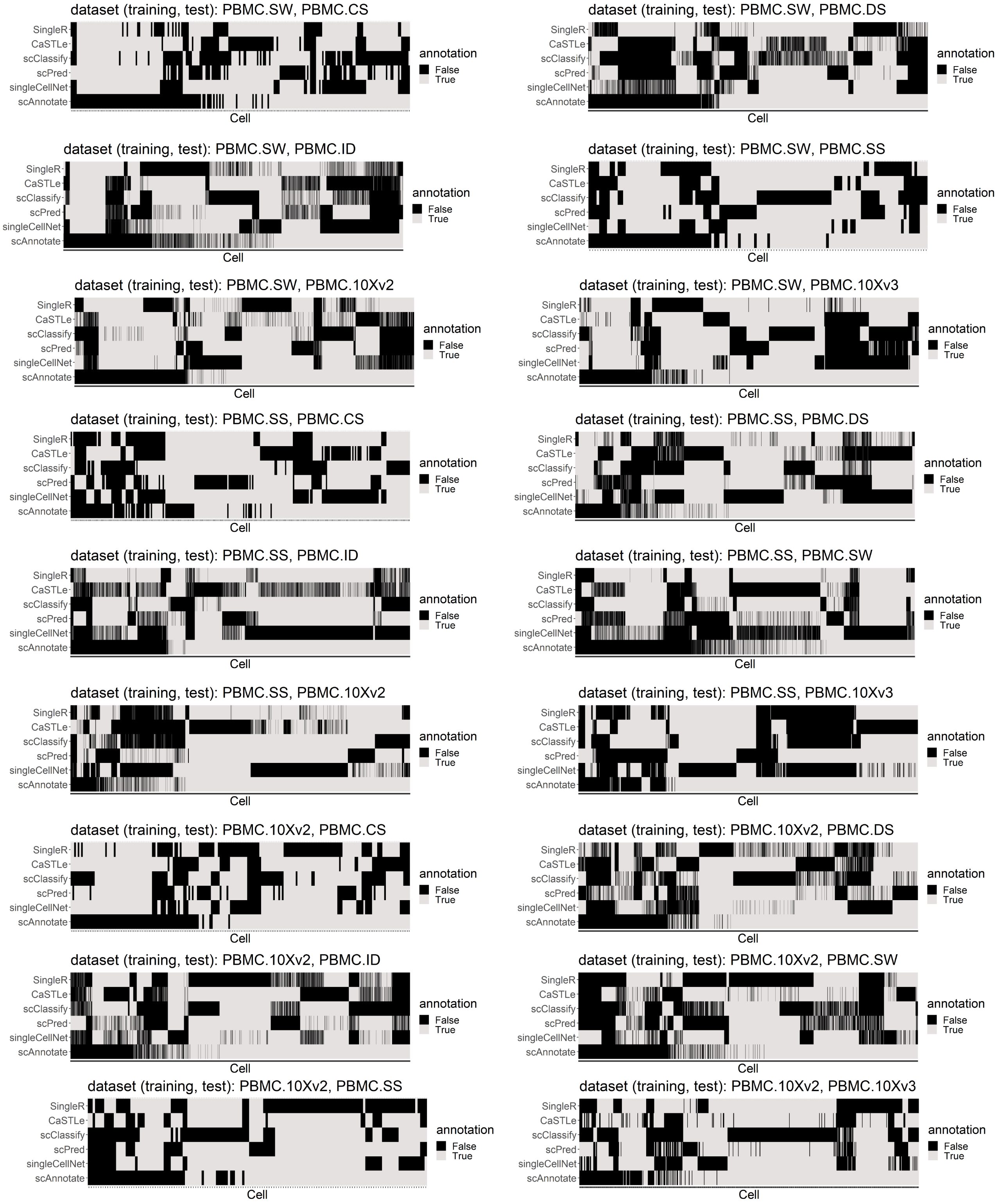

Figure S2. The mosaic plot shows the cells of each dataset that are inconsistently annotated by the top six benchmarked methods.

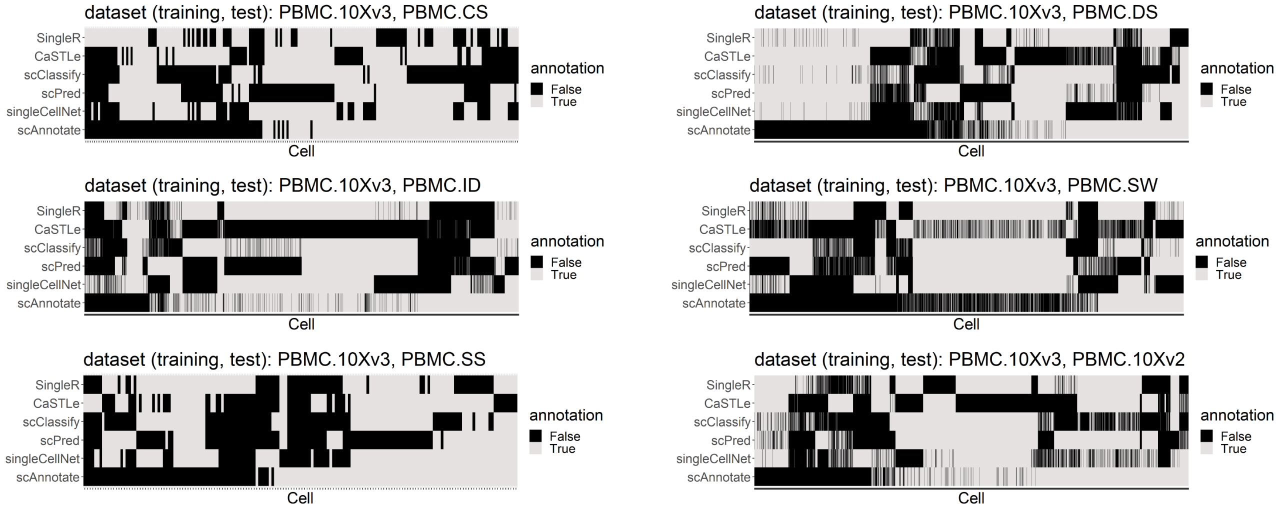

Figure S3. The mosaic plot shows the cells of each dataset that are inconsistently annotated by the top six benchmarked methods.

References

[1] Rish I, et al. An empirical study of the naive Bayes classifier. In: IJCAI 2001 workshop on empirical methods in artificial intelligence. vol. 3; 2001. p. 41-6.

[2] Ding J, Adiconis X, Simmons SK, Kowalczyk MS, Hession CC, Marjanovic ND, et al. Systematic comparative analysis of single cell RNA-sequencing methods. bioRxiv. 2019. Available from: `https://www.biorxiv.org/content/10.1101/632216v2`.

[3] Boufea K, Seth S, Batada NN. scID uses discriminant analysis to identify transcriptionally equivalent cell types across single-cell RNA-seq data with batch effect. IScience. 2020;23(3):100914.

[4] Lin Y, Cao Y, Kim HJ, Salim A, Speed TP, Lin DM, et al. scClassify: sample size estimation and multiscale classification of cells using single and multiple reference. Molecular systems biology. 2020;16(6):e9389.

[5] Tan Y, Cahan P. SingleCellNet: a computational tool to classify single cell RNA-Seq data across platforms and across species. Cell systems. 2019;9(2):207-13.

[6] Alquicira-Hernandez J, Sathe A, Ji HP, Nguyen Q, Powell JE. scPred: accurate supervised method for cell-type classification from single-cell RNA-seq data. Genome biology. 2019;20(1):1-17.

[7] Lieberman Y, Rokach L, Shay T. CaSTLe–classification of single cells by transfer learning: harnessing the power of publicly available single cell RNA sequencing experiments to annotate new experiments. PloS one. 2018;13(10):e0205499.

[8] Aran D, Looney AP, Liu L, Wu E, Fong V, Hsu A, et al. Reference-based analysis of lung single-cell sequencing reveals a transitional profibrotic macrophage. Nature immunology. 2019;20(2):163-72.

[9] de Kanter JK, Lijnzaad P, Candelli T, Margaritis T, Holstege FC. CHETAH: a selective, hierarchical cell type identification method for single-cell RNA sequencing. Nucleic acids research. 2019;47(16):e95-5.

[10] Kiselev VY, Yiu A, Hemberg M. scmap: projection of single-cell RNA-seq data across data sets. Nature methods. 2018;15(5):359-62.

[11] Tian L, Dong X, Freytag S, Cao KAL, Su S, JalalAbadi A, et al. Benchmarking single cell RNA-sequencing analysis pipelines using mixture control experiments. Nature methods. 2019;16:479-87.

[12] Tasic B, Yao Z, Graybuck LT, Smith KA, Nguyen TN, Bertagnolli D, et al. Shared and distinct transcriptomic cell types across neocortical areas. Nature. 2018;563:72-8.

[13] Hodge RD, Bakken TE, Miller JA, Smith KA, Barkan ER, Graybuck LT, et al. Conserved cell types with divergent features in human versus mouse cortex. Nature. 2019;573:61-8.

[14] Baron M, Veres A, Wolock SL, Faust AL, Gaujoux R, Vetere A, et al. A single-cell transcriptomic map of the human and mouse pancreas reveals inter-and intra-cell population structure. Cell systems. 2016;3(4):346-60.

[15] Abdelaal T, Michielsen L, Cats D, Hoogduin D, Mei H, Reinders MJT, et al. A comparison of automatic cell identification methods for single-cell RNA sequencing data. Genome biology. 2019;20(194).

[1]Department of Mathematics & Statistics, University of Victoria, Victoria, V8P 5C2, Canada, [2]Department of Mathematics & Statistics, University of Saskatchewan, Saskatoon, S7N 5C9, Canada, * Corresponding authors: Xuekui Zhang (xuekui@uvic.ca) and Li Xing (li.xing@math.usask.ca
